# Optimizing patient outcomes: a comprehensive evaluation of protocolized sedation in intensive care settings: a systematic review and meta-analysis

**DOI:** 10.1186/s40001-024-01839-y

**Published:** 2024-04-24

**Authors:** Fredy Leonardo Carreño Hernandez, Maria Valentina Stozitzky Ríos, Yenny Rocio Cardenas Bolivar, Jorge Iván Alvarado Sánchez

**Affiliations:** 1 Clinical Research, Universidad de los Andes, School of medicine, Bogotá, Colombia; 2 Clinical Research, Fundación Santa Fe de Bogotá, Bogotá, Colombia; 3 Intensive Care Unit, Hospital Universitario Fundación Santa Fe de Bogotá, Bogotá, Colombia; 4 Intensive Care Unit, Hospital Universitario Fundación Santa Fe de Bogotá, Universidad Nacional de Colombia, Bogotá, Colombia

**Keywords:** Sedation, Protocolized sedation, ICU, Ventilation

## Abstract

**Introduction:**

Amidst the routine utilization of protocolized sedation in ventilated ICU patients, existing management guidelines exhibit a lack of unanimous recommendations for its widespread adoption. This study endeavors to comprehensively assess the effectiveness and safety of protocolized sedation in critically ill ventilated patients.

**Objective:**

The primary objective of this study was to systematically review and conduct a meta-analysis of clinical trials comparing protocolized sedation with standard management in critically ill ventilated patients. Key outcomes under scrutiny include ICU and hospital mortality, ventilation days, duration of ICU stay, and incidents of self-extubation. The evaluation incorporates the Risk of Bias 2 (RoB2) tool to assess the quality of included studies. Data analysis utilizes a random-effects model for relative risk (RR) and mean differences. Subgroup analysis categorizes sedation protocols into algorithmic or daily interruption, addressing potential heterogeneity. Additionally, a GRADE evaluation is performed to ascertain the overall certainty of the evidence.

**Results:**

From an initial pool of 1504 records, 10 studies met the inclusion criteria. Protocolized sedation demonstrated a reduced RR for mortality (RR: 0.80, 95% CI 0.68–0.93, *p* < 0.01, *I*^2^ = 0%) and a decrease in ventilation days (mean difference: − 1.12, 95% CI − 2.11 to − 0.14, *p* = 0.03, *I*^2^ = 84%). Furthermore, there was a notable reduction in ICU stay (mean difference: − 2.24, 95% CI − 3.59 to − 0.89, *p* < 0.01, *I*^2^ = 81%). However, incidents of self-extubation did not exhibit a significant difference (RR: 1.20, 95% CI 0.49–2.94, *p* = 0.69, *I*^2^ = 35%). Subgroup analyses effectively eliminated heterogeneity (*I*^2^ = 0%), and the GRADE evaluation yielded moderate results for mortality, ventilation days, and ICU duration.

**Conclusion:**

Protocolized sedation, whether implemented algorithmically or through daily interruption, emerges as a safe and effective approach when compared to standard management in ventilated ICU patients. The findings from this study contribute valuable insights to inform evidence-based practices in sedation management for this critical patient population.

**Supplementary Information:**

The online version contains supplementary material available at 10.1186/s40001-024-01839-y.

## Introduction

In the realm of critical care, sedative agents play an indispensable role in addressing pain, managing agitation, ensuring proper sleep, and, most crucially, facilitating effective ventilation in patients undergoing invasive procedures. This challenge is further complicated by the intricacies brought on by the COVID-19 pandemic. Sedation in the context of invasive ventilation introduces a host of complications, ranging from difficulties with self-extubation and ventilator-acquired pneumonia (VAP) to prolonged stays in the intensive care unit (ICU) [1].

To navigate these challenges, diverse strategies have been developed, encompassing the selection of appropriate sedatives, and refining their administration methods and frequencies. One particularly promising approach is protocolized sedation, involving meticulous titration of a patient's sedation levels, which has demonstrated efficacy in reducing the duration of ventilator support [2]. Protocolized sedation can be further delineated into algorithmic protocols, daily interruptions, or a combination thereof.

However, systematic assessments of protocolized sedation in ventilated patients aimed at averting adverse events have faced hurdles. A glaring example is the 2018 Pain, Agitation/Sedation, Delirium, Immobility (Rehabilitation/Mobilization), and Sleep (Disruption) (PADIS) guideline. This guideline lacks consensus regarding the use of protocolized sedation in sedated patients to mitigate adverse events in ventilated patients [3]. This uncertainty is rooted in a systematic review with meta-analysis conducted in 2015 by Minhas, which aimed to establish whether protocolized sedation could curtail ventilation time, mortality rates, the incidence of self-extubating, or ICU length of stay [4]. However, Minhas’ analysis only yielded conclusive evidence for the latter parameter. Additionally, new studies have been published, and it is necessary to obtain current evidence on this important topic.

This study aims to evaluate the impact of protocolized sedation on clinical outcomes in critically ill patients receiving mechanical ventilation in the intensive care unit (ICU). It involves comparing the effects of protocolized sedation, implemented through careful titration, with conventional sedation lacking a specific titration protocol. Primary outcome includes patient mortality and secondary outcomes include incidence of ventilator-associated pneumonia (VAP), self-extubation rates, and both duration of ICU stay and ventilation days.

## Methodology

### Protocol

This meta-analysis adheres to the Preferred Reporting Items for Systematic Reviews and Meta-Analysis (PRISMA) recommendations [5]. The comprehensive and predefined protocol has been registered with PROSPERO™ under the registration number CRD42023392876 (https://www.crd.york.ac.uk/prospero/display_record.php?RecordID=392876).

### Search strategy and data extraction

A search was conducted on MEDLINE, COCHRANE, and EMBASE up to November 2022, along with clinical trial databases such as ClinicalTrials.gov and the International Clinical Trials Registry Platform (ICTRP) of the World Health Organization. The search was focused on records in Spanish and English as outlined in the PROSPERO protocol. Two authors (F, L, C; V, S) independently reviewed titles and potentially eligible abstracts using the Rayyan© tool. Discrepancies were resolved through consensus among the authors.

### Inclusion criteria

Studies meeting the following PICOS criteria were included:

- Participants: Intensive care unit patients requiring invasive ventilation for any reason.
- Interventions encompassed protocolized sedation, wherein nurses or physicians employed a titration strategy. Protocolized sedation referred to the application of standardized approaches for managing sedation in ICU patients, such as utilizing a sedation algorithm or implementing daily sedation interruption. In contrast, usual care involved no protocolized, discretion-based sedation management, where clinicians directed the sedation process.
- Comparator: Protocolized sedation vs usual care.
- Outcomes:
  - Primary: Mortality.
  - Secondary: VAP, self-extubating, both ventilation and ICU length of days.
- Study types: Randomized clinical trials with or without blinding and concealment.

Articles meeting any of the following criteria were excluded: language other than Spanish or English, inability to access the full text, only measures of association without raw data, case reports or series, observational and quasiexperimental studies, and abstract congress.

### Risk of bias assessment

Two authors (F, L, C; V, S) conducted a risk of bias assessment using the Risk of Bias 2 (RoB2) tool [6]. Discrepancies were resolved through consensus among the authors. This tool was employed to evaluate potential biases in the included clinical trials, focusing on aspects such as randomization, intervention deviation, data loss, outcome measurement errors, and selectivity in data reporting**.**

### Data items

Data extraction was manually performed by two researchers (F, L, C; V, S) and recorded in an Excel© sheet. Discrepancies were resolved through consensus among the authors. Extracted variables included primary author, publication year, country, sedatives used, exclusion criteria, total patients, intervention and comparator patient counts, mortality, mean age, frequency of comorbidities, surgical and trauma frequencies, ventilation cause, self-extubating frequency, days in ICU, and days on ventilation.

### Statistical analysis

A rigorous meta-analysis was performed using Review Manager 5 (RewMan5©) software. The analysis employed appropriate statistical methods for both dichotomous and continuous outcomes. For dichotomous outcomes such as VAP, mortality, and self-extubating, the weighted relative risk (RR) was calculated. This involves pooling data from individual studies and calculating a summary estimate of the effect size, considering both the sample size and effect size of each study. The random-effects model was applied to account for potential heterogeneity across studies. For continuous variables, such as ICU length of stay and days on ventilation, standardized mean differences was calculated. This involves comparing the mean outcomes between groups while standardizing for the scale of measurement. The random-effects model was utilized for this analysis. In cases where data are reported in medians with interquartile ranges or other nonmean formats, a conversion to means was performed using Sean McGrath’s Box‒Cox method [7]. This approach ensures consistency in data representation, allowing for appropriate inclusion in the meta-analysis.

Heterogeneity among studies was assessed using the Cochran *Q* statistic and the *I*^2^ index. A significant *Q* statistic or high *I*^2^ value may indicate substantial heterogeneity, prompting further investigation [8]. Subgroup analyses were conducted between studies that utilized daily interruption or algorithmic sedation as a form of sedation protocols. This would help explore potential variations in outcomes related to different sedation strategies.

### Additional assessment

Sensitivity analysis was performed to assess the robustness of the findings. This involves systematically varying aspects of the analysis, such as excluding studies with a high risk of bias, to evaluate the impact on the overall results. Publication bias was evaluated using funnel plots. These graphical representations will assess the symmetry of the distribution of effect sizes, aiding in the detection of potential bias toward the publication of studies with significant results.

To assess the certainty of evidence for each outcome, the GRADE approach [9] was followed. Certainty levels (high, medium, low, or very low) was assigned based on judgments about the randomization process, intervention deviation, data loss, outcome measurement, selection of reported results, and overall judgment. The results were summarized in an evidence table.

## Results

A total of 2243 records were initially identified through multiple search engines. Subsequently, 1504 where left as for abstract review by eliminating 736 duplications. Only 45 of those abstracts underwent full-text assessment, with 10 ultimately being chosen for inclusion in this review, which also involved a meta-analysis. All 10 selected articles utilized the RASS scale for sedative titration and 9 out 10 studies incorporated benzodiazepines into their pharmacological regimens.

**Fig. 1:**
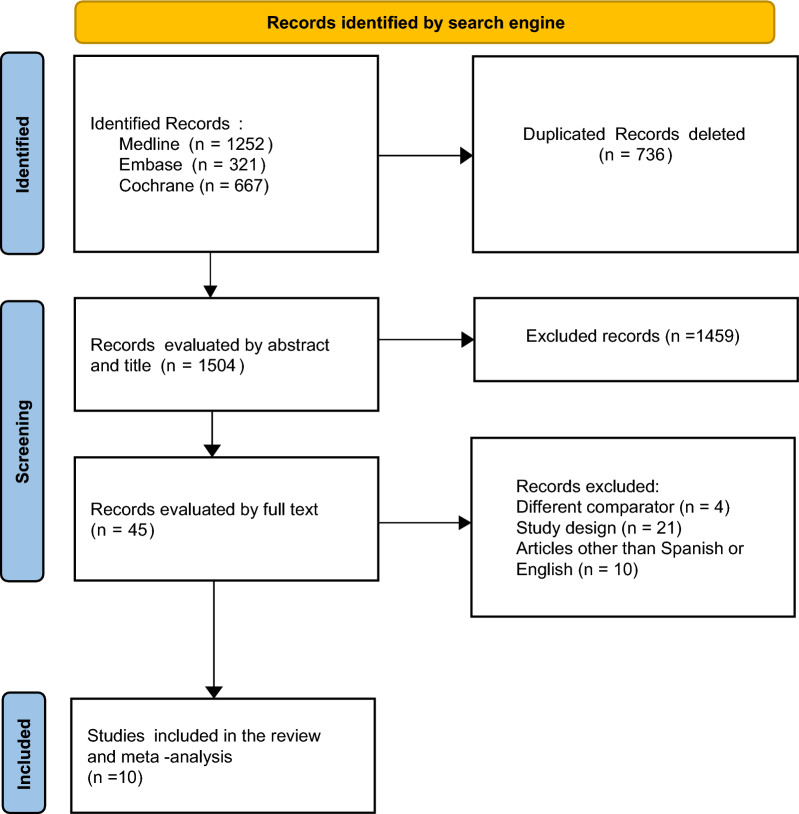
PRISMA flowgram

Among the reported studies, only 2 did not provide information on the reasons for patient intubation, and none reported occurrences of ventilator-associated pneumonia. Additionally, all excluded patients had experienced resuscitation from cardiorespiratory arrest, displayed neurological deficits, needed muscle blockers, were in an imminent death situation, or were anticipated to spend less than 24 h in the ICU. The screening process is visually represented in Fig. 1, detailed characteristics of the studies can be found in Table 1 and Additional file 1: Table S1.

**Table 1:** General characteristic of included studies

Author—year	Country	Sedatives used	*n* Intervention/comparison	Sedation protocol methodology	Criteria exclusion
Taran—2019 [10]	Iran	Fentanyl, methadone, midazolam, haloperidol, morphine, thiopental	39 40	Algorithm	Extubating and alertness in the first 24 h, modification or, suspension of a drug prescribed by the attending physician, transfer to the operating room for surgery, Glasgow criteria below 5 and initiation of continuous infusion of sedative
Brook—1999 [11]	USA	Propofol, haloperidol, fentanyl, lorazepam, diazepam	162 159	Algorithm	Were temporarily (< 24 h) admitted to the medical intensive care unit from a surgical ward while awaiting a surgical intensive care unit bed
Weisbrodt—2011 [12]	USA	Fentanyl, midazolam, propofol	26 24	Daily interruption	Had a neurological or neurosurgical diagnosis at admission, drug overdose, could not understand English, as do not resuscitated orders
Anifantaki—2009 [13]	Greece	Remifentanil, propofol, midazolam	49 48	Daily interruption	Pregnancy, transfer to the ICU after resuscitation after cardiac arrest or initiation of sedative infusion in another hospital
Bucknall—2008 [14]	Australia	Fentanyl, midazolam, diazepam, haloperidol, morphine, vecuronium	153 159	Algorithm	Cardiac surgery and those admitted to the ICU who had been on the study previously
Tanios—2019 [15]	USA	Fentanyl, midazolam, propofol	28–31 29	Algorithm and algorithm + daily interruption	Cardiac arrest, acute neurologic injury, acute alcohol withdrawal, conditions that precludes sedation, or continuous IV opioid or sedative use ≥ 24 h
Shehabi—2013 [16]	Australia	Dexmedetomidine, propofol	21 16	Algorithm	Minors, pregnancy, neurological injury, overdose, burn injury, acute liver failure, dementia, shock
Kress—2000 [17]	USA	Midazolam, propofol	68 60	Daily interruption	Pregnancy, transfer from an outside institution where sedatives had already been administered, and admission after resuscitation from cardiac arrest
Girard—2008 [18]	USA	Benzodiazepines, opiates, propofol	168 167	Daily interruption	Cardiopulmonary arrest, continuous mechanical ventilation for 2 weeks or more, moribund state, withdrawal from life support, profound neurological deficits, or current enrollment in another clinical trial
Mansouri—2013 [19]	Iran	Morphine, fentanyl, sufentanil, midazolam, propofol, haloperidol	95 105	Algorithm	Had less than 24 h of ICU stay, were expected to die in less than 48 h, had received muscle relaxants, received anticonvulsant medication for seizures, had psychological illness, or had paralysis of the upper extremities or immobilization in a cast

### General characteristics of the studies included

The majority of the studies were conducted in the USA, and they encompassed various types of sedative drugs. Among these studies, five specifically assessed sedation algorithms as a form of protocolized sedation. In contrast, four studies implemented daily sedation interruption, and another utilized both daily sedation interruption and a sedation algorithm as part of their approaches to protocolized sedation.

### Risk of bias

Of the 10 included studies, none had a high risk of bias for any component, only one had some concerns of bias in the component in the randomization process, and 7 studies had some concerns of bias in the missing data by nonreporting component. In the other components, all studies presented a low risk of bias. The complete evaluation with the RoB2 tool can be found in Table 2.

**Table 2:** Risk of bias 2 evaluation

Author—year	Randomization process	Deviation from the proposed intervention	Lost data	Outcome measurement	Selection of reported results	Overall judgment
Taran—2019 [10]	?	✓	✓	✓	✓	✓
Brook—1999 [11]	✓	✓	?	✓	✓	✓
Weisbrodt—2011 [12]	✓	✓	✓	✓	✓	✓
Anifantaki—2009 [13]	✓	✓	?	✓	✓	✓
Bucknall—2008 [14]	✓	✓	?	✓	✓	✓
Tanios (A)—2019 [15]	✓	✓	?	✓	✓	✓
Tanios (B)—2019 [15]	✓	✓	?	✓	✓	✓
Shehabi—2013 [16]	✓	✓	?	✓	✓	✓
Kress—2000 [17]	✓	✓	?	✓	✓	✓
Girard—2008 [18]	✓	✓	✓	✓	✓	✓
Mansouri—2013 [19]	✓	✓	?	✓	✓	✓

✓ = low risk; ? = some concerns

### Synthesis of results

In terms of mortality, a statistically significant reduction was observed with protocolized sedation compared to usual ICU management, as indicated by an RR of 0.80 [95% CI 0.68–0.93, *I*^2^ = 0%; *p* < 0.01]. Both sedation protocols involving daily interruption (RR = 0.79, 95% CI 0.63–0.99, *I*^2^ = 0%, *p* = 0.04) and algorithm-based sedation (RR = 0.82, 95% CI 0.66–1.03, *I*^2^ = 0%, *p* = 0.09) contributed to decreased mortality. Conversely, in the context of self-extubation events, protocolized sedation did not show a significant decrease compared to usual ICU management, with an RR of 1.20 [95% CI 0.49–2.94, *I*^2^ = 35%; *p* = 0.69].

**Fig. 2:**
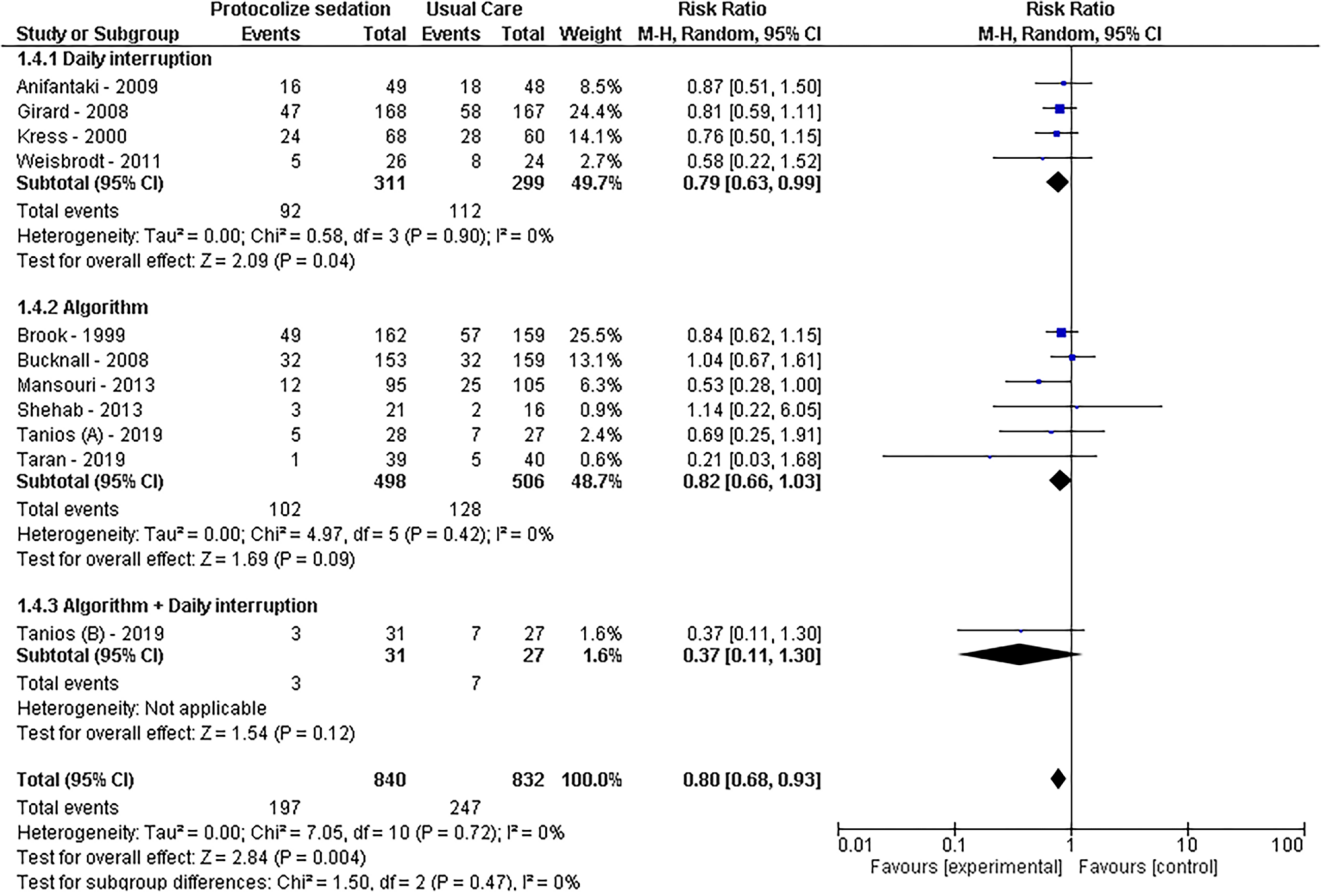
Mortality forest plot by protocolized sedation methodology

**Fig. 3:**
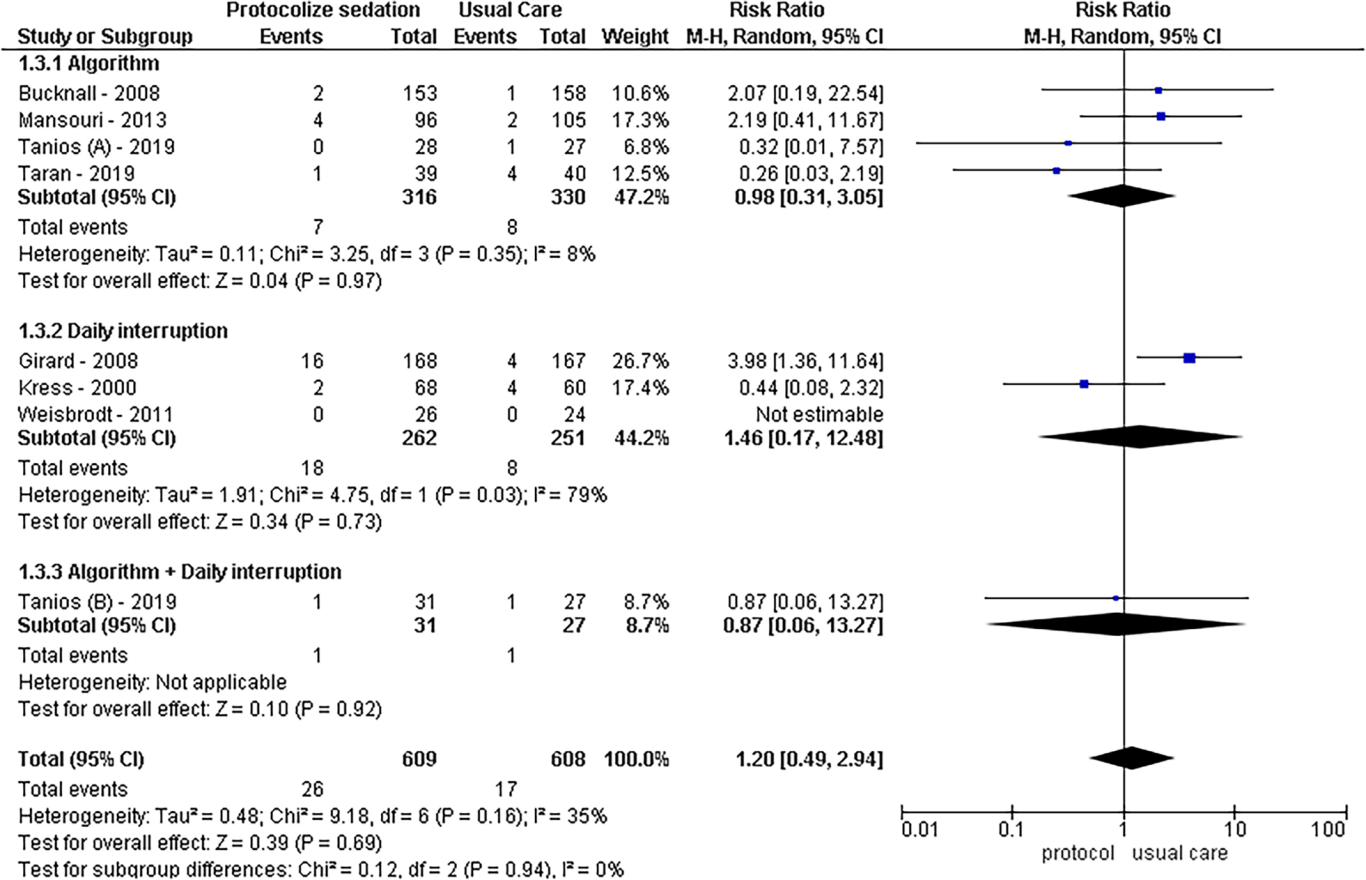
Self-extubation forest plot by protocolized sedation methodology

**Fig. 4:**
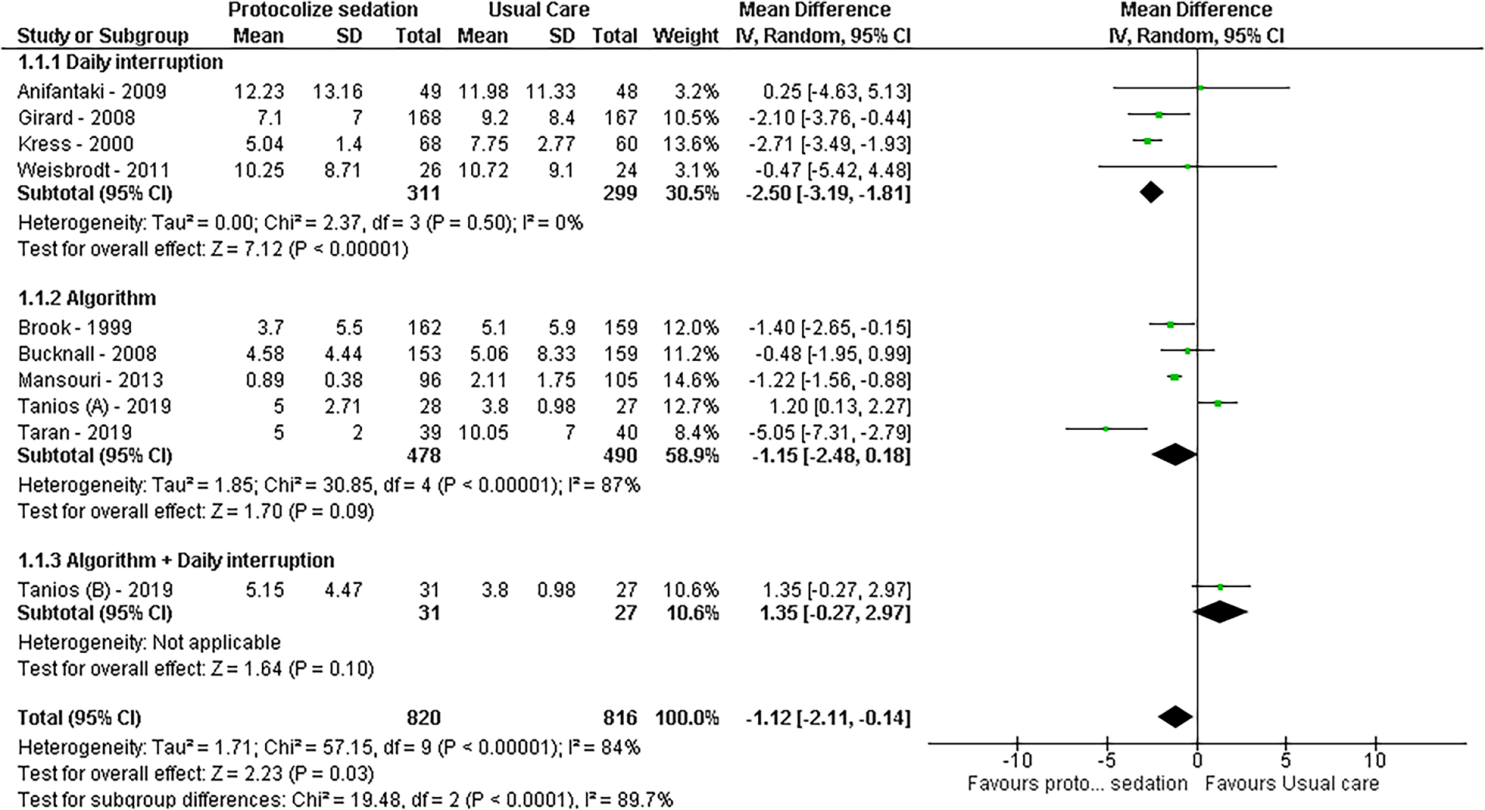
Days in ventilation forest plot by protocolized sedation methodology

**Fig. 5:**
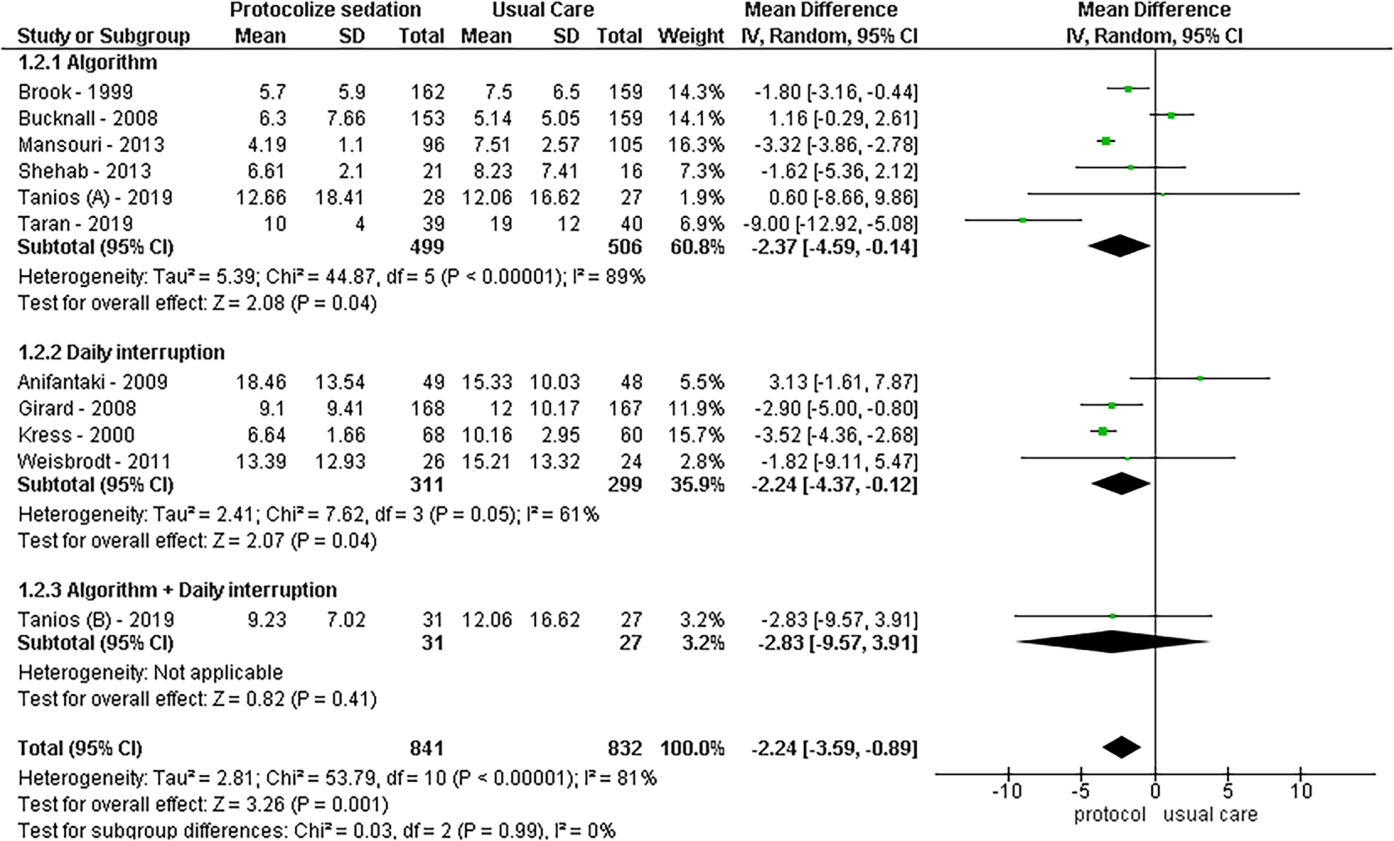
Days in ICU forest plot by protocolized sedation methodology

Regarding ventilation-related outcome, analysis of nine studies revealed that protocolized sedation led to a reduction in ventilation days by 1.12 days [95% CI − 2.11 to − 0.14, *I*^2^ = 89%; *p* = 0.03]. Notably, daily interruption demonstrated a more pronounced effect, showing a decrease of 2.50 days [95% CI − 3.19 to − 1.81, *I*^2^ = 0%; *p* < 0.01], while algorithm-based sedation was not statistically significant, resulting in 1.15 fewer days [95% CI − 2.48 to − 0.18, *I*^2^ = 87%; *p* = 0.9]. Furthermore, for the duration of ICU stay, protocolized sedation, both by daily interruption and algorithm, exhibited a reduction of 2.24 days [95% CI − 3.59 to − 0.89, *I*^2^ = 81%; *p* < 0.01], with subgroup analysis reducing heterogeneity to *I*^2^ = 0%. The results are visually represented in Figs. 2, 3, 4, and 5.

### Risk of bias across studies

**Fig. 6:**
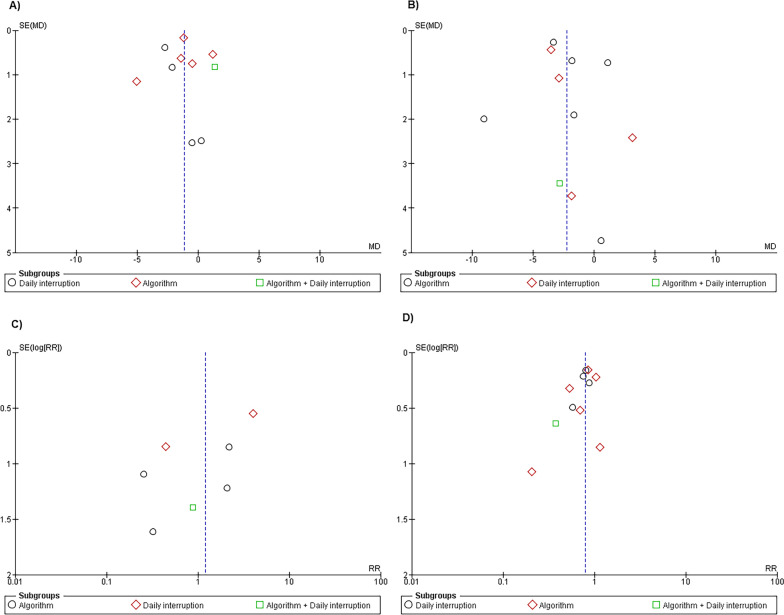
Funnel plot. **A** Ventilation days. **B** Days on ICU. **C** Self-extubation. **D** Mortality

No publication bias was identified, as evidenced by the absence of asymmetry in funnel plots for all evaluated outcomes, as illustrated in Fig. 6A–D. This indicates that the findings presented in this review are unlikely to be distorted by selective reporting, enhancing the robustness and reliability of the reported results.

### Additional assessment

#### Sensitivity analysis

Given the considerable prevalence of bias related to lost data, a sensitivity analysis was conducted with a subset of 3 studies. The results for self-extubating events (RR = 1.19, 95% CI 0.08–17.22) and reduced days of ventilation (RR = 2.95, 95% CI − 5.39 to − 0.51) and ICU stay (RR = 4.82, 95% CI − 9.36 to − 0.28) maintained a consistent direction of effect, although with variations in magnitude compared to the overall results. Mortality, however, exhibited a nonsignificant RR of 0.70 (95% CI 0.48–1.02). A secondary sensitivity analysis, excluding the only study with concerns regarding the randomization process, indicated a shift in the ventilation day differences (RR = − 0.78, 95% CI − 1.74 to 0.18), with no statistically significant variations identified.

#### GRADE assessment

Based on the comprehensive assessment of the identified risks, predominantly low in various components with some concerns related to missing data, coupled with remarkable consistency in the results and the potential explanation of heterogeneity by the type of protocolized sedation, moderate evidence was established for the effectiveness of protocolized sedation in reducing mortality, ventilation days, and ICU stay. In contrast, due to significant inconsistency in the outcomes and the inability to elucidate heterogeneity through subgroup analysis, protocolized sedation was determined to have very low evidence concerning the occurrence of self-extubating events. A detailed summary of these findings is presented in Table 3.

**Table 3:** GRADE assessment

No. of studies	Study design	Risk of bias	Certainty assessment		Vagueness	Other considerations	No. of patients		Effect		Certainty	Importance
			Inconsistency	Indirect evidence			Protocolized sedation	Usual care	Relative (95% CI)	Absolute (95% CI)		
Mortality												
10	Randomized trials	Serious	Not serious	Not serious	Not serious	None	260/1037 (25.1%)	335/1058 (31.7%)	OR 0.80 (0.70 to 0.92)	46 less per 1000 (from 72 to 18 less)	⨁⨁⨁◯ Moderate	CRITICAL
Ventilation days												
8	Randomized trials	Serious	Not serious	Not serious	Not serious	None	820	816	–	MD 1.12 days less (2.11 less to 0.14 less)	⨁⨁⨁◯ Moderate	IMPORTANT
Length of days in ICU												
10	Randomized trials	Serious	Not serious	Not serious	Not serious	None	1038	1058	–	MD 2.3 days less (3.58 less to 1.02 less)	⨁⨁⨁◯ Moderate	IMPORTANT
Self-extubation												
7	Randomized trials	Serious	Very serious	Not serious	Very serious	None	26/609 (4.3%)	17/608 (2.8%)	OR 1.20 (0.49 to 2.94)	5 more for 1000 (from 14 less to 50 more)	⨁◯◯◯ Very low	IMPORTANT

*CI* confidence interval, *MD* mean difference, *OR* odds ratio

## Discussion

In this comprehensive systematic review with meta-analysis, we scrutinized ten studies that compared the efficacy of protocolized sedation against standard care in ventilated ICU patients. Our analysis revealed substantial advantages associated with protocolized sedation, leading to a notable reduction in ventilation and ICU days and a concurrent improvement in safety indicated by lower mortality rates. Despite potential biases related to data loss, the GRADE evaluation moderately recommends the adoption of protocolized sedation over usual care for these three critical outcomes.

Furthermore, our subgroup analyses, distinguishing between daily interruption and algorithmic continuation of protocolized sedation, consistently demonstrated benefits over usual care. However, the analysis of the reduction in self-extubating rates remains inconclusive due to insufficient data for comprehensive evaluation. Notably, the challenge of assessing the specific intervention combining both daily interruption and algorithmic protocols arises from the limited number of studies with this unique cohort.

### Association with previous studies

Our systematic review and meta-analysis significantly build upon the foundation laid by Minhas et al. in 2015 [4]. By identifying two new studies and incorporating two previously overlooked studies, our findings reveal a decrease in mortality associated with protocolized sedation, contrasting with Minhas’ earlier report. This discrepancy underscores the importance of our review’s inclusivity, enabling the detection of nuanced differences between the intervention and the comparator. Similar trends were observed in the reduction in ICU length of stay and ventilation days, reinforcing the efficacy of protocolized sedation.

An intriguing aspect of our analysis involved subgroup assessments based on the type of protocolized sedation employed. The distinctions between algorithmic and daily interruption protocols, while both adhering to specific guidelines, became evident in the respective outcomes, showcasing variations in relative risk (RR) and mean differences for each type of protocolized sedation.

It is noteworthy that, despite the absence of blinding in the intervention across all studies, the impact on outcomes was minimal. This can be attributed to the study's focus on objective and concrete outcomes, resulting in a low risk of bias.

### Significance and implications

The implications of our systematic review on the management of ventilated patients are profound, particularly considering the previous absence of a recommendation for protocolized sedation in the PADIS 2018 guidelines due to insufficient evidence. With the presented findings and the anticipated release of the updated PADIS 2023 guide, we anticipate influencing forthcoming recommendations. Moreover, we advocate for the PADIS 2023 guide to differentiate between algorithmic protocolized sedation and daily interruption, facilitating more precise and tailored recommendations.

The consistency in exclusion criteria across the studies allows us to identify specific patient populations for whom this intervention might be less effective. Understanding the reasons behind patient intubation further enables the identification of those who could benefit most from protocolized sedation.

Looking forward, conducting clinical trials directly comparing different types of protocolized sedation, including combined approaches, becomes imperative. Our systematic review predominantly compared protocolized sedation against continuous infusion or daily sedation management, preventing a conclusive determination of the superiority of one protocolized sedation method over another due to the lack of direct comparative studies.

## Conclusions

In conclusion, protocolized sedation demonstrates a significant reduction in mortality, ventilation days, and ICU stay compared to standard sedation management for intubated ICU patients. However, distinctions between algorithmic protocolized sedation and diurnal interruption exist, emphasizing the need for specific clinical trials directly comparing these methods. The insights provided by our study contribute to advancing evidence-based practices in sedation management for ventilated ICU patients.

### Supplementary Information

**Additional file 1: Table S1.** General characteristic of included studies.

## Data Availability

The datasets used and/or analyzed during the current study are available from the corresponding author upon reasonable request.
